# Editorial: Unravelling *T. cruzi* Biology

**DOI:** 10.3389/fcimb.2020.00382

**Published:** 2020-07-28

**Authors:** Martin Craig Taylor, Noelia Lander, Nobuko Yoshida

**Affiliations:** ^1^Faculty of Infectious and Tropical Diseases, London School of Hygiene and Tropical Medicine, London, United Kingdom; ^2^Center for Tropical and Emerging Global Diseases, University of Georgia, Athens, GA, United States; ^3^Departamento de Microbiologia, Imunologia e Parasitologia, Escola Paulista de Medicina, Universidade Federal de São Paulo, São Paulo, Brazil

**Keywords:** *Trypanosoma cruzi*, Chagas disease, parasite-host interaction, gene function, CRISPR/Cas9 technique

*Trypanosoma cruzi*, a protozoan parasite that causes Chagas disease, affects an estimated 8 million people, mainly in Latin America, but also in many developed countries where the disease is not endemic. Options for drug treatment are limited and not fully effective, especially in the chronic phase of the disease. The genetic complexity of *T. cruzi*, with a number of gene families with hundreds of members and genes of unknown function, in addition to the absence of efficient techniques for genetic manipulation before the CRISPR era, have contributed to the slow progress in understanding the biology of this parasite. The aim of this Research Topic is to provide an overview of the current knowledge on diverse aspects of *T. cruzi* biology. We received contributions ranging from molecular and cellular approaches to evaluate gene function and analyze parasite organelles, to studies on parasite-host interactions, signal transduction during infection, alternative pathways for identification of new drug targets, pathology of Chagas disease in animal model and *T. cruzi* biology in the wild environment, as well as in different stages of the parasite's life cycle.

Several studies in this topic assessed *T. cruzi* gene functions using the CRISPR/Cas9 system. Using a CRISPR/Cas9-riboswitch-based method, Lander et al. succeeded in the downregulation at the mRNA level of genes encoding glycoprotein 72 (*TcGP72*) and vacuolar proton pyrophosphatase (*TcVP1*), as proof of concept. They found that TcVP1 is not essential for the viability of epimastigotes or the infective stages of *T. cruzi*, although it is important for normal growth of epimastigotes in rich medium, and for trypomastigote invasion of host cells. Also using CRISPR/Cas9, Malvezzi et al. generated *T. cruzi* devoid of *Tc*K1, an ortholog of the protein kinase named general control non-repressible 2 (GCN2) that phosphorylates the eukaryotic initiation factor 2 alpha subunit (eIF2α). Under starvation, TcK1 depleted cells showed reduced accumulation of stress RNA granules, independently of eIF2α phosphorylation. In absence of *Tc*K1, metacyclogenesis increased, but the trypomastigotes were less infective to mammalian host cells. Regarding *T. cruzi* structure, Chasen et al. used a combination of CRISPR/Cas9-mediated endogenous tagging, fluorescently labeled overexpression constructs and endocytic assays, to identify the first known cytostome-cytopharynx complex (SPC) targeted protein (CP1), which co-localizes with endocytosed protein and disassembles in infective forms of *T. cruzi*. Two additional proteins that target to SPC (CP2 and CP3) were also identified. In addition, they defined the location of a region adjacent to the SPC entrance, known as the pre-oral ridge (POR), which lies between the entrance of the flagellar pocket and the cytostome. CRISPR/Cas9 technology was also used by Grazielle-Silva et al. to investigate the involvement of DNA mismatch repair (MMR) component MSH6 in the oxidative stress response in *T. cruzi*, by generating MSH6 null mutants. The loss of one or two alleles of *T. cruzi msh6* resulted in increased susceptibility to H_2_O_2_ exposure, besides impaired MMR. They confirmed that MSH2 associated with MSH6 is a central component of the MMR pathway responsible for the recognition and correction of base mismatches that occur during DNA replication and recombination. In their review on RNA binding proteins (RBPs) and gene expression regulation in *T. cruzi*. Romagnoli et al. also presented data on downregulation of three RBPs, by using the CRISPR/Cas9 technique. RBP silencing affected cell division. References were made to the characterization of messenger ribonucleoprotein (mRNP) particles, which can be organized into larger complexes forming stress RNA granules, depending on RBPs. They suggest that protein composition of the mRNPs as well as the localization and fate of mRNAs, and consequently of the genes expressed, depend on RBPs, which allow the coordinated expression of mRNAs encoding proteins that are related in function, resulting in the formation of post-transcriptional operons. All of these studies highlight the impact that CRISPR/Cas9 technology has had on *T. cruzi* research.

Ramirez discussed the co-evolution of telomeres, subtelomeres, and the trans- sialidase type II (TSII) family, and the role that these regions may have played in shaping *T. cruzi*'s genome. It is suggested that these regions, together with retrotransposon elements, promote the generation of genetic variability, and that members of the TS II family positioned at the subtelomeres co-evolved to be part of the transition to the telomeric repeat. This review also proposed that double strand breaks introduced in the subtelomeres by retrotransposon nucleases are repaired by homologous recombination, and when the repair includes non-homologous chromatids there is a possibility to generate gene variants.

To survive the inimical conditions *T. cruzi* encounters in the insect vector and the mammalian host, the parasite has to adapt to distinct microenvironments, where nutrient availability, osmolarity, ionic concentrations, and pH vary significantly. Mesías et al. reviewed the current knowledge of the oxidant environment experienced by *T. cruzi* in the insect and mammalian hosts, and the molecular strategies exploited by the parasite to deal with oxidative stress. *T. cruzi* utilizes a network of antioxidant enzymes to control the cytotoxic effects of reactive oxygen species (ROS) and reactive nitrogen species (RNS) to which they are exposed in the triatomine and the mammalian hosts. In their review, Melo et al. discussed how environmental factors, such as temperature, availability of nutrients and consequent oxidative and osmotic stresses may alter *T. cruzi* development in the triatomine insect. Replication of epimastigotes and differentiation into metacyclic trypomastigotes are affected by these factors. Another study in this Research Topic investigated *T. cruzi* adaptation to different environments. Barrera et al. aimed at characterizing an ion channel that plays a critical role in parasite survival. They identified and characterized the expression pattern and function of a novel calcium-activated potassium channel (TcCAKC), which resides in the plasma membrane of all three life stages of *T. cruzi*. They generated TcCAKC null parasites, which showed impaired growth, decreased production of trypomastigotes and slower intracellular replication. Epimastigotes lacking the channel had significantly lower cytosolic calcium, hyperpolarization, changes in intracellular pH, and increased proton extrusion rate.

*Trypanosoma. cruzi* infection may induce the inflammatory process that ultimately lead to myocardiopathy. Extracellular vesicles (EVs) shed by trypomastigote forms of *T. cruzi* could be involved in promoting inflammation. Cronemberger-Andrade et al. have found that *T. cruzi*-infected macrophages shed EVs that interact with Toll-like-receptor 2 (TLR2) and stimulate the translocation of NF-κB, inducing expression of proinflammatory cytokines (TNF-α, IL-6, and IL-1β), and STAT-1 and STAT-3 signaling pathways. In addition, EVs from parasites or from *T. cruzi*-infected macrophages enhanced host cell invasion. Nisimura et al. re-analyzed previous microarray dataset from four different *T. cruzi* strains, in order to better understand the transcriptomic impact that each strain has on JAK-STAT signaling and cell cycle pathways. They reported that *T. cruzi* strains differentially alter the expression of JAK-STAT signaling, whose activation may lead to muscle cell hypertrophy, and distinctly modulate cell cycle pathways.

Identification of targets for new drugs to treat Chagas disease is an important issue. Moreover, the ability of *T. cruzi* to establish dormancy associated with resistance to drug treatment has been recently reported. Using different *T. cruzi* DTUs during their replicative epimastigote and amastigote stages, Resende et al. investigated the influence of recombinational processes to induce dormancy in *T. cruzi*. For both epimastigote and amastigote forms, the number of dormant cells was higher in hybrid than in non-hybrid strains. Treatment of *T. cruzi* with gamma radiation, which generates DNA lesions repaired by homologous recombination, resulted in increased percentage of dormancy. In parasites harboring single knockout for RAD51, there was a significant reduction in the number of dormant cells. Altogether, these data suggest the existence of an adaptive difference among *T. cruzi* strains to generate dormant cells, and that homologous recombination could be important for dormancy in this parasite.

Potential therapeutic targets for treatment of *T. cruzi* infection have been identified by exploiting the differences between the mechanisms involved in intracellular Ca^2+^ signaling in humans and trypanosomatids. Benaim et al. reviewed data that support targeting Ca^2+^ homeostasis as a strategy against Chagas disease by giving examples of reported drugs with trypanocidal effects. In another review, Quiñones et al. discussed the potential of glycosomal enzymes, which are essential for the trypanosomatid viability, as drug targets. Biochemical and proteomic analysis of glycosomes have revealed the integration of glycosomes in overall *T. cruzi* metabolism. In these authors' view, the differences in human and trypanosomatid proteins make a variety of glycosomal enzymes promising drug targets. It is also referred that selective, potent inhibitors of some glycosomal proteins have been demonstrated to interfere with metabolic processes.

Carvalho et al. conducted a robust echocardiographic evaluation of left ventricular (LV) function in dogs chronically infected with *T. cruzi*. Parameters analyzed included end-systolic volume (ESV), end-diastolic volume (EDV), ejection fraction (EF), and fractional shortening (FS). A significant LVEF and FS reduction was observed in infected animals compared to controls. The authors concluded that the canine model of chronic chagasic cardiomyopathy (CCC) mimics human disease, reproducing the percentage of individuals that develop heart failure during the chronic infection.

In an integrative review, Martinez et al. discussed the current state of diagnosis and prognosis of Chagas disease, the treatment for the disease, approaches to improve treatment, as well as host factors and parasite susceptibility and resistance to drug. Prediction of disease outcome and determining whether and when treatment of infection may be necessary, still remains as a prospect to be fulfilled.

*T. cruzi* is primarily a parasite of wild mammals. Jansen et al. reviewed *T. cruzi* biology in the natural environment. They discussed among other topics: *T. cruzi* genotypes and ecology, transmission in nature, *T. cruzi* biology in opossums, mixed infections and the recent outbreaks of acute Chagas disease by oral infection.

Overall, this Research Topic brings together an important collection of articles focused on fundamental aspects of *T. cruzi* biology, using state-of-the-art technology to elucidate the function of proteins and the metabolic pathways to which they belong; as well as reviewing the current literature involving the main features of the parasite that causes Chagas disease.

## Author Contributions

All authors listed have made a substantial, direct and intellectual contribution to the work, and approved it for publication.

## Conflict of Interest

The authors declare that the research was conducted in the absence of any commercial or financial relationships that could be construed as a potential conflict of interest.

